# Analysis of the Taxonomy and Pathogenic Factors of *Pectobacterium aroidearum* L6 Using Whole-Genome Sequencing and Comparative Genomics

**DOI:** 10.3389/fmicb.2021.679102

**Published:** 2021-07-02

**Authors:** Peidong Xu, Huanwei Wang, Chunxiu Qin, Zengping Li, Chunhua Lin, Wenbo Liu, Weiguo Miao

**Affiliations:** ^1^Key Laboratory of Green Prevention and Control of Tropical Plant Diseases and Pests, Ministry of Education, College of Plant Protection, Hainan University, Haikou, China; ^2^School of Life Sciences, Hainan University, Haikou, China

**Keywords:** genome sequence, taxonomy, pathogenic gene, *Pectobacterium aroidearum*, comparative genomics

## Abstract

Soft rot pectobacteria are devastating plant pathogens with a global distribution and a broad host range. *Pectobacterium aroidearum* L6, previously isolated from leaves of *Syngonium podophyllum*, is a pectolytic bacterial pathogen that causes typical soft rot on *S. podophyllum*. There is a shortage for genome data of *P. aroidearum*, which seriously hinders research on classification and pathogenesis of *Pectobacterium*. We present here the complete genome sequence of *P. aroidearum* L6. The L6 strain carries a single 4,995,896-bp chromosome with 53.10% G + C content and harbors 4,306 predicted protein-coding genes. We estimated *in silico* DNA–DNA hybridization and average nucleotide identity values in combination with the whole-genome-based phylogeny from 19 *Pectobacterium* strains including *P. aroidearum* L6. The results showed that L6 and PC1 formed a population distinct from other populations of the *Pectobacterium* genus. Phylogenetic analysis based on 16S rRNA and genome sequences showed a close evolutionary relationship among *Pectobacterium* species. Overall, evolutionary analysis showed that L6 was in the same branch with PC1. In comparison with 18 *Pectobacterium* spp. reference pathogens, strain L6 had 2,712 gene families, among which 1,632 gene families were identified as orthologous to those strains, as well as 1 putative unique gene family. We discovered 478 genes, 10.4% of the total of predicted genes, that were potentially related to pathogenesis using the Virulence Factors of Pathogenic Bacteria database. A total of 25 genes were related to toxins, 35 encoded plant cell-wall degrading enzymes, and 122 were involved in secretion systems. This study provides a foundation for a better understanding of the genomic structure of *P. aroidearum* and particularly offers information for the discovery of potential pathogenic factors and the development of more effective strategies against this pathogen.

## Introduction

Soft rot *Pectobacteriaceae* are considered to be one of the top ten most important agricultural phytopathogens (Mansfield et al., 2012). The family *Pectobacteriaceae* consisted of *Pectobacterium* spp. and *Dickeya* spp., formerly characterized as pectinolytic *Erwinia* spp. (Pérombelon, 2002; Charkowski et al., 2012; Adeolu et al., 2016). They cause destructive soft rot on a variety of field crops, fruits, ornamentals, and vegetables, including the staple food crop potato (Toth et al., 2003, 2011; Ma et al., 2007). *Pectobacterium* was first established in 1945 (Waldee, 1945; Ma et al., 2007). However, the classification of *Pectobacterium* spp. is not clear. Hauben et al. (1998) established the genus *Pectobacterium* that included three species and five subspecies: *P. cacticidum, P. chrysanthemi, P. cypripedii, P. carotovorum* subsp. *atrosepticum, P. carotovorum subsp. betavasculorum, P. carotovorum* subsp. *carotovorum, P. carotovorum* subsp. *odoriferum*, and *P. carotovorum* subsp. *wasabiae*. Subsequently, three subspecies of *P. carotovorum* were elevated into the species level, namely *P. atrosepticum, P. odoriferum*, and *P. wasabiae* (Gardan et al., 2003; Duarte et al., 2004; Nykyri et al., 2012). The classification of the genus *Pectobacterium* has been subjected to wide revision over the last decade, and it is likely that some of the genome-sequenced strains have been incorrectly assigned to *P. carotovorum* (Gardan et al., 2003; Khayi et al., 2016; Pritchard et al., 2016; Zhang et al., 2016). For instance, *P. carotovorum* subsp. *carotovorum* tends to serve as a catchall for pectobacteria isolates differing from the specific descriptions of the other pectobacteria taxa, and *P. aroidearum* was classified as a novel species in 2013 (Nabhan et al., 2013). The genome-sequenced strain PC1 (formerly classified as *P. carotovorum* subsp. *carotovorum*) is actually *P. aroidearum* under the new classification (Nabhan et al., 2013).

In the age of genomics, the *Pectobacterium* genus has been subjected to revision based on the development of bioinformatics. Several pectolytic bacterial strains were thought to belong to a novel *Pectobacterium* species after several taxonomic analyses including 16S rRNA gene sequence, DNA–DNA hybridization (DDH), genomics, and comparative genomics. These include *P. actinidiae* (Koh et al., 2012), *P. polaris* (Dees et al., 2017), *P. peruviense* (Waleron et al., 2018), *P. zantedeschiae* (Waleron et al., 2019), *P. punjabense* (Sarfraz et al., 2018, 2020), and *P. aroidearum* (Nabhan et al., 2013). The species of the genus Pectobacterium has 18 of child taxa with a validly published with correct name and four proposed species not yet validated based on The List of Prokaryotic names with Standing in Nomenclature^1^ (Table 1; Adeolu et al., 2016; Parte et al., 2020). Eleven species had complete genome data, and 7 had not complete assembly based on the National Center for Biotechnology Information (NCBI) genome database^2^ (Table 1). Only strain PC1 of *P. aroidearum* has its whole genome sequenced. Thus, there is a shortage of whole-genome data for *P. aroidearum*.

**Table 1:** Nomenclatural status and type strain of genome data of the *Pectobacterium* genus.

Name	Nomenclatural status	Origin	Type strain of genome data
*Pectobacterium actinidiae*	Validly published	Portier et al. (2019)	KKH3
*Pectobacterium aquaticum*	Validly published	Pédron et al. (2019)	No complete assembly
*Pectobacterium aroidearum*	Validly published	Nabhan et al. (2013)	PC1(formerly classified as P. carotovorum, not corrected in the NCBI database)
*Pectobacterium atrosepticum*	Validly published	Gardan et al. (2003)	JG1008, 21A
*Pectobacterium betavasculorum*	Validly published	Gardan et al. (2003)	No complete assembly
*Pectobacterium brasiliense*	Validly published	Portier et al. (2019)	SX309, 1,692
*Pectobacterium cacticida*	Validly published	Hauben et al. (1998)	No complete assembly
*Pectobacterium carnegieana*	Validly published	Brenner et al. (1973)	No complete assembly
*Pectobacterium carotovorum*	Validly published	Waldee (1945)	JR1.1, 67
*Pectobacterium fontis*	Validly published	Oulghazi et al. (2019)	No complete assembly
*Pectobacterium odoriferum*	Validly published	Portier et al. (2019)	JK2.1, BC S7
*Pectobacterium parmentieri*	Validly published	Khayi et al. (2016)	HC, RNS 08–42-1A
*Pectobacterium parvum*	Validly published	Pasanen et al. (2020)	No complete assembly
*Pectobacterium polaris*	Validly published	Dees et al. (2017)	PZ1, NIBIO1006
*Pectobacterium polonicum*	Validly published	Waleron et al. (2019)	No complete assembly
*Pectobacterium punjabense*	Validly published	Sarfraz et al. (2018)	SS95
*Pectobacterium versatile*	Validly published	Portier et al. (2019)	14A, 3–2
*Pectobacterium wasabiae*	Validly published	Gardan et al. (2003)	CFBP 3304
*Pectobacterium delphinii*	Not validly published	Waldee (1945)	
*Pectobacterium melonis*	Not validly published	Waldee (1945)	
*Pectobacterium peruviense*	Not validly published	Waleron et al. (2018)	
*Pectobacterium zantedeschiae*	Not validly published	Waleron et al. (2019)	

Currently, no methods and chemicals are effective in controlling *Pectobacterium* disease or preventing the spread of these pathogens (Zhang et al., 2017). In addition, planting patterns and storage conditions are not applicable for control of the disease (Yaganza et al., 2014). Bacterial strain L6, isolated from *Syngonium podophyllum* soft rot samples in Hainan Province, was recognized as *P. aroidearum* (Xu et al., 2020). There is a shortage for whole-genome data of *P. aroidearum*, which seriously hinders research on classification and pathogenesis of *Pectobacterium*. Genome comparison revealed that most virulence genes are highly conserved in the *Pectobacterium* strains, especially for the key virulence determinants involved in the biosynthesis of extracellular enzymes and secretion systems (Li et al., 2019). The functional genomics methods are the effective ways to elucidate that this pathogen interacts with plants and causes disease (Toth et al., 2015). In this study, we sequenced the whole genome of *P. aroidearum* L6. Then, we compared it with genome analyses of 18 *Pectobacterium* reference strains. Furthermore, the genome annotation and comparative genomics analysis provided a foundation for a better understanding of the genomic structure of *P. aroidearum* and particularly offered information for the discovery of potential pathogenic factors and the development of more effective strategies against this pathogen.

## Materials and Methods

### Strain and Type Strain Genome Sequences

Strain L6 was previously isolated from *S. podophyllum* soft rot samples in a plant nursery in the Haidian campus of Hainan University, Haikou, Hainan Province, China, in July 2019. Samples were collected from symptomatic *S. podophyllum* for bacterial isolation. Internal fragments containing symptomatic tissues were transferred to 1 mL of sterile distilled water, after 20 min, rinsed with 70% alcohol and sterile distilled water, and cultured onto Luria-Bertani (LB) medium for 48 h at 28°C to differentiate and characterize the bacterial pathogen. A total of 10 bacterial colonies were isolated from infected tissues. The isolated colonies were subcultured until the pure culture of the suspected bacterium was obtained. Two representative isolates (L5 and L6) were selected for further tests and one isolate preserved in Key Laboratory of Green Prevention and Control of Tropical Plant Diseases and Pests (Hainan University), Ministry of Education as *P. aroidearum* L6.

The pathogenicity of *P. aroidearum* L6 was previously reported (Xu et al., 2020). The putative pathogen was re-inoculated to confirm its pathogenicity in the incubator and field on the leaves of *S. podophyllum*. Typical symptoms of soft rot were observed 12–24 h after inoculation. *P. aroidearum* was re-isolated from a diseased leaf, fulfilling Koch’s postulates. L6 was grown on LB broth for 12–24 h at 28°C. And genomic DNA of L6 was extracted by the Bacteria Genomic DNA Extraction Kit (Tiangen Biotech Co. Ltd., Beijing, China). Successful extraction of genomic DNA was confirmed by 0.8% agarose gels and quantified by Nanodrop ND-2000 (Thermo Fisher Scientific, United States). All complete genome sequences of *Pectobacterium* were retrieved from NCBI. Type strain genome sequences of 11 species which had complete assembly genome data were used, including *P. aroidearum* PC1 (GCF_000023605.1), *P. carotovorum* subsp. *carotovorum* JR1.1 (NZ_CP034237.1), *P. carotovorum* subsp. *carotovorum* 67 (NZ_CP034211.1), *P. atrosepticum* JG10-08 (NZ_CP007744.1), *P. atrosepticum* 21A (NZ_CP009125.1), *P. brasiliense* SX309 (NZ_CP020350.1), *P. brasiliense* 1,692 (NZ_CP047495.1), *P. odoriferum* JK2.1 (NZ_CP034938.1), *P. odoriferum* BC S7 (NZ_CP009678.1), *P. polaris* PZ1 (NZ_CP046377.1), *P. polaris* NIBIO1006 (NZ_CP017481.1), *P. actinidiae* KKH3 (NZ_JRMH01000001.1), *P. wasabiae* CFBP 3304 (NZ_CP015750.1), *P. parmentieri* HC (NZ_CP046376.1), *P. parmentieri* RNS 08–42-1A (NZ_CP015749.1), *P. versatile* 14A (NZ_CP034276.1), *P. versatile* 3–2 (NZ_CP024842.1), and *P. punjabense* SS95 (NZ_CP038498.1).

### Genome Sequencing and Assembly

The *P. aroidearum* L6 genome was sequenced using a PacBio RS II platform and Illumina HiSeq 4,000 platform. For Illumina HiSeq sequencing, the fragments of 470 bp (with the approximate insert size of 350 bp) from adaptor-ligated DNA were recovered according to standard protocols. The libraries with different indices were multiplexed and loaded on an Illumina HiSeq instrument. Cutadapt (v1.9.1; Martin, 2011) was employed for quality control, and reads whose base groups have quality value below 20 at both ends, sequences containing more than 10% N base, or less than 75 bp in length were removed. The Illumina data were used for estimate and correction. Four SMRT cells zero-mode waveguide (a nano-optical device used to confine light to a small observation volume) arrays of sequencing were used in the PacBio platform to generate the subreads set. PacBio subreads (length < 1 kb) were removed. The program pbdagcon^3^ was used for self-correction. Draft genomic unitigs, which are uncontested groups of fragments, were assembled using the Celera Assembler (Myers et al., 2000) against a high-quality corrected circular consensus sequence subreads set. To improve accuracy of the genome sequences, the Genome Analysis Toolkit^4^ (Mckenna et al., 2010) and SOAP tool packages (SOAP2, SOAPsnp, SOAPindel; Li et al., 2013) were used to make single-base corrections. To trace the presence of any plasmid, the filtered Illumina reads were mapped using SOAP to the bacterial plasmid database.^5^

### Genome Component Prediction

Gene prediction was performed on the L6 genome assembly using NCBI Prokaryotic Genome Annotation Pipeline^6^ (Tatusova et al., 2016; Haft et al., 2018; Li et al., 2021). The tRNA, rRNA, and sRNA recognition made use of tRNAscan-SE (Lowe and Eddy, 1997), RNAmmer (Lagesen et al., 2007), and the Rfam database (Gardner et al., 2009). Tandem repeats annotation was obtained using the Tandem Repeat Finder^7^ (Wirawan et al., 2010), and the minisatellite DNA and microsatellite DNA were selected based on the number and length of repeat units. The Genomic Island Suite of Tools GIST v1.0 was used for genomic island analysis with Island Path-DIOMB, SIGI-HMM, and Island Picker method (Hasan et al., 2012). Prophage regions were predicted using the PHAge Search Tool web server^9^ (Zhou et al., 2011) and CRISPR identification using CRISPR Finder (Grissa et al., 2007).

### Phylogenetic Analysis

The maximum-likelihood (ML) phylogenetic analysis was inferred with FastME 2.1.6.1 including SPR postprocessing (Lefort et al., 2015) from the Genome BLAST Distance Phylogeny approach (GBDP) distances calculated using default settings from 16S rRNA gene sequences and genome sequences. Branch support was inferred from 1,000 pseudo-bootstrap replicates each. The trees were rooted at the midpoint (Farris, 1972) and visualized with PhyD3 (Kreft et al., 2017).

### Average Nucleotide Identity and *in* *silico* DNA–DNA Hybridization Analysis

Pairwise comparison of their Average Nucleotide Identity (ANI) was based on BLAST+ (ANIb) from JSpeciesWS^10^ (Richter et al., 2015), while *in silico* DNA–DNA Hybridization (*is*DDH) was conducted using GBDP by the Type (Strain) Genome Server^11^ (Meier-Kolthoff and Göker, 2019). One-hundred distance replicates were calculated each. The DDH values and confidence intervals were calculated using the recommended settings of the Genome-to-Genome Distance Calculator (GGDC 2.1) for GGDC formula 2 (Meier-Kolthoff et al., 2013).

### Comparative Genomics

*Pectobacterium aroidearum* L6 was compared with reference 18 *Pectobacterium* strains by using their genome sequence and gene sequence. BLAST core/pan genes of the six strains were clustered using CD-HIT (Fu et al., 2012) rapid clustering of similar proteins software with a threshold of 50% pairwise identity and 0.7 length difference cutoff in amino acids. Gene families were constructed using genes of L6 and reference strains. We carried out gene family TreeFam clustering treatment for the alignment results by Hcluster_sg software (Maqbool and Babri, 2007) and multiple sequence alignment with the clustered gene family using Muscle software (Edgar, 2004a,b). The phylogenetic tree was constructed using multiple sequence alignment results based on Muscle by the TreeBeST (Nandi et al., 2010) using the ML method.

### Gene Annotation and Protein Classification

The function annotation is accomplished by analysis of protein sequences. We align genes with databases to obtain the highest quality corresponding annotations. Seven databases were used for general function annotation: Kyoto Encyclopedia of Genes and Genomes (KEGG; Kanehisa et al., 2016), Clusters of Orthologous Groups (COG; Galperin et al., 2015; Makarova et al., 2015), Non-Redundant Protein Database (NR), Swiss-Prot (UniProt Consortium, 2015), Gene Ontology (GO; Ashburner et al., 2000; Philip et al., 2014), TrEMBL (Apweiler et al., 2004), and Evolutionary Genealogy of Genes (EggNOG: Non-supervised Orthologous Groups; Huerta-Cepas et al., 2016) databases. Four databases were used for pathogenicity and drug resistance analysis. Virulence factors and resistance genes were identified based on the core dataset in Virulence Factors of Pathogenic Bacteria (VFDB; Chen et al., 2016) and the Antibiotic Resistance Genes Database (Liu and Pop, 2009); the other two databases were Pathogen Host Interactions (PHI; Winnenburg et al., 2006) and Carbohydrate-Active enZYmes Database (CAZy; Levasseur et al., 2013). Type III secretion system (T3SS) effector proteins were detected using EffectiveT3 (Vargas et al., 2012).

## Results

### Genomic Features Among *P. aroidearum* L6 and Reference Strains

**Figure 1:**
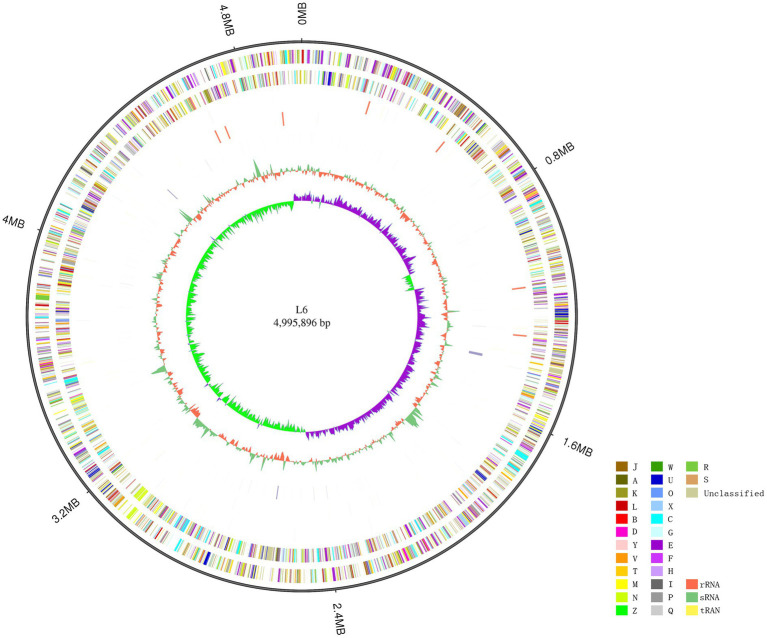
Circular representation of the *Pectobacterium aroidearum* L6 genome. From outer to inner: first circle is genome size; second and third circles are forward and reverse strand gene, respectively, colored according to cluster of COG classification (A, RNA processing and modification; B, chromatin structure and dynamics; C, energy production and conversion; D, cell cycle control, cell division, and chromosome partitioning; E, amino acid transport and metabolism; F, nucleotide transport and metabolism; G, carbohydrate transport and metabolism; H, coenzyme transport and metabolism; I, lipid transport and metabolism; J, translation, ribosomal structure, and biogenesis; K, transcription; L, replication, recombination, and repair; M, cell wall/membrane/envelope biogenesis; N, cell motility; O, posttranslational modification, protein turnover, and chaperones; P, inorganic ion transport and metabolism; Q, secondary metabolites biosynthesis, transport, and catabolism; R, general function prediction only; S, function unknown; T, signal transduction mechanisms; U, intracellular trafficking, secretion, and vesicular transport; V, defense mechanisms; W, extracellular structures; X, mobilome: prophages, transposons; Y, nuclear structure; Z, cytoskeleton); fourth and fifth circles are ncRNA (yellow indicates tRNA, orange indicates rRNA, and green indicates sRNA) and repeat, respectively; seventh circle is GC content (green indicates greater than average value, and orange indicates less than average value); and eighth circle is GC-SKEW (GC-SKEW = (G − C)/(G + C), purple indicates >0, and green indicates <0).

The *P. aroidearum* L6 genome was sequenced using a PacBio RS II platform and Illumina HiSeq 4,000 platform. The raw data were filtered, and this generated 1, 274 Mb of clean data. The reads were assembled into a genome and then assessed. We obtained a genome size of 4,995,896 bp, which consisted of a circular chromosome with no plasmid (Figure 1). GC-Depth analysis was performed on the assembly results to show the assembly length (4,995,896 bp), coverage length (4,995,089), coverage (99.98%), reads usage percent (95.3%), and depth (260) of *P. aroidearum* L6. The average G + C content of the genome was 53.10%. A total of 4,306 putative protein-coding sequences, with total length of 4,298,622 bp (86.04% of total genome length; Table 2) and average length of 998.29 bp, were annotated on the *P. aroidearum* L6 genome. The genome encoded 77 tRNA operons and 40 sRNA genes. In addition, a total of 22 rRNA operons were present on the chromosome: eight 5S rRNAs, seven 16S rRNAs, and seven 23S rRNAs.

**Table 2:** Genomic features of the *P. aroidearum* L6 genome and comparison with genomes of reference strains.

	Genome size (bp)	G+C content (mol%)	Gene number	Clustered gene number	Number of rRNAs	Number of tRNAs	Family number	Unique family number
*P. aroidearum* L6	4,995,896	53.1	4,306	4,209	22	77	2,712	1
*P. aroidearum* PC1	4,862,913	51.9	4,201	4,132	22	78	2,670	1
*P. carotovorum* subsp. *carotovorum* JR1.1	4,872,902	52.0	4,086	4,019	22	76	2,667	0
*P. carotovorum* subsp. *carotovorum* 67	4,909,824	51.3	3,532	3,436	22	75	2,361	8
*P. atrosepticum* JG10-08	5,004,926	51.1	4,245	4,215	22	76	2,805	0
*P. atrosepticum* 21A	4,991,806	51.1	4,323	4,296	22	77	2,850	0
*P. brasiliense* SX309	4,966,299	52.2	4,209	4,137	22	76	2,733	1
*P. brasiliense* 1,692	4,851,982	52.2	4,145	4,044	22	77	2,649	0
*P. odoriferum* JK2.1	4,997,932	51.5	4,356	4,158	22	77	2,786	4
*P. odoriferum* BC S7	4,933,575	51.8	3,912	3,830	22	77	2,570	2
*P. polaris* PZ1	4,994,870	51.0	4,115	3,923	22	77	2,621	8
*P. polaris* NIBIO1006	4,826,824	52.0	4,088	4,010	22	77	2,645	1
*P. actinidiae* KKH3	4,068,673	51.5	4,152	4,079	21	76	2,624	1
*P. wasabiae* CFBP 3304	5,043,228	50.6	4,369	4,203	22	78	2,805	2
*P. parmentieri* HC	5,208,618	50.4	4,494	4,366	22	77	2,935	1
*P. parmentieri* RNS 08–42-1A	5,030,841	50.4	4,423	4,343	22	77	2,922	4
*P. versatile* 14A	4,997,114	51.8	4,304	4,250	22	77	2,778	2
*P. versatile* 3–2	4,975,878	51.8	4,266	4,191	22	78	2,737	1
*P. punjabense* SS95	4,793,778	50.7	4,152	4,036	22	76	2,639	0

*The genes were taken from reference genome as gene pool. The blast results were filtered by their length and identity. Gene number, the gene number in each strain; Clustered gene number, the gene number that can be clustered in gene family; Family number, the gene family number in strain; Unique family number, the unique gene family number in strain*.

### Phylogenetic Analysis of *P. aroidearum* L6

**Figure 2:**
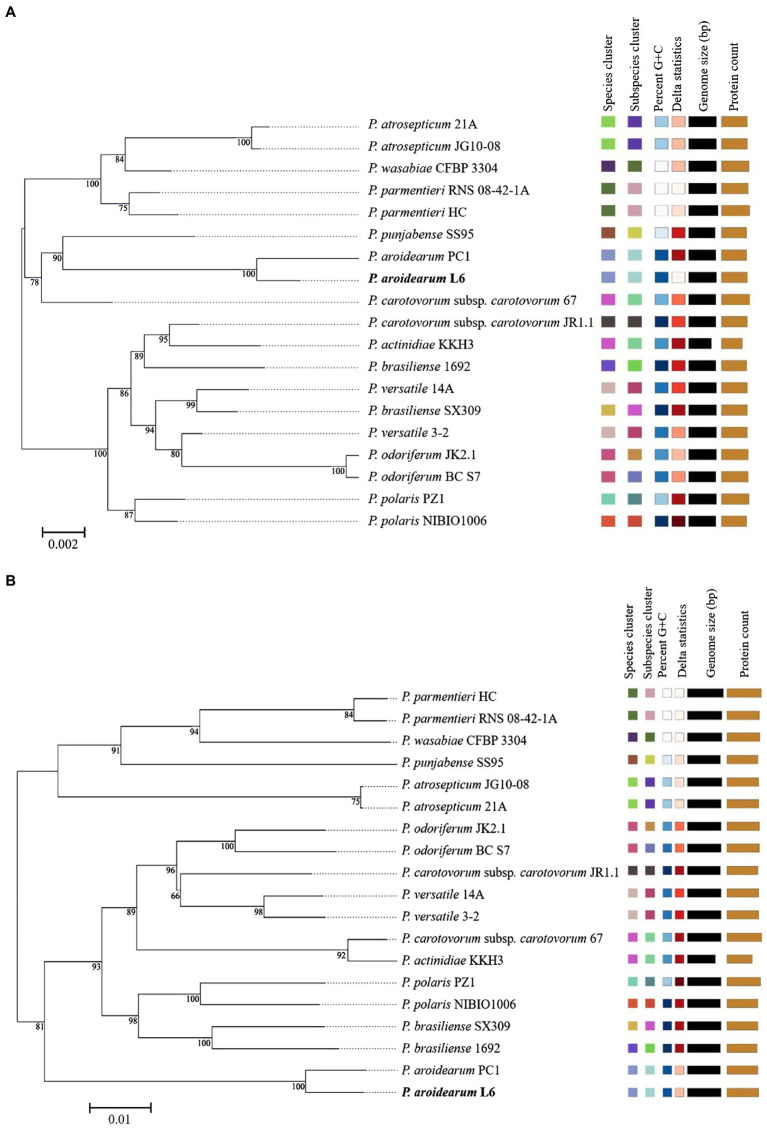
ML phylogenetic analysis for *P. aroidearum* L6 and reference strains. **(A)** Phylogenetic tree drawn using the 16S rRNA of *Pectobacterium* spp. **(B)** Phylogenetic tree drawn using the genomes of *Pectobacterium* spp. Bootstrap values are indicated in % of repetitions.

The 16S rRNA is the most useful and is commonly used as a molecular clock in the systematic classification of bacteria. Its evolution has good clock properties, being highly conserved in structure and function, and can well reflect the differences between different bacteria (Coenye and Vandamme, 2010). Thus, the accessibility of a large quantity of completely sequenced bacterial genomes allows the speedy and reliable determination of intragenomic sequence heterogeneity of 16S rRNA genes. The phylogenetic tree was inferred with FastME 2.1.6.1 from GBDP distances calculated from *Pectobacterium* species 16S rRNA and genome sequences. The branch lengths were scaled in terms of GBDP distance formula d5. The numbers on branches are GBDP pseudo-bootstrap support values >60% from 1,000 replications, with an average branch support of 91.1%. The delta value was 0.315 based on 16S rRNA and 0.156 based on genome sequences. A phylogram based on computing of the 16S rRNA suggested a close relationship between both *P. aroidearum* L6 and PC1 genomes (Figure 2A). The phylogenetic relationships among *P. aroidearum* L6 and reference strains were determined based on genome sequence results (Figure 2B). The whole-genome-based phylogenetic tree showed that L6 was most closely related to *P. aroidearum* PC1.

### Assessment of Taxonomy of *Pectobacterium* Species Using *is*DDH and ANI

**Figure 3:**
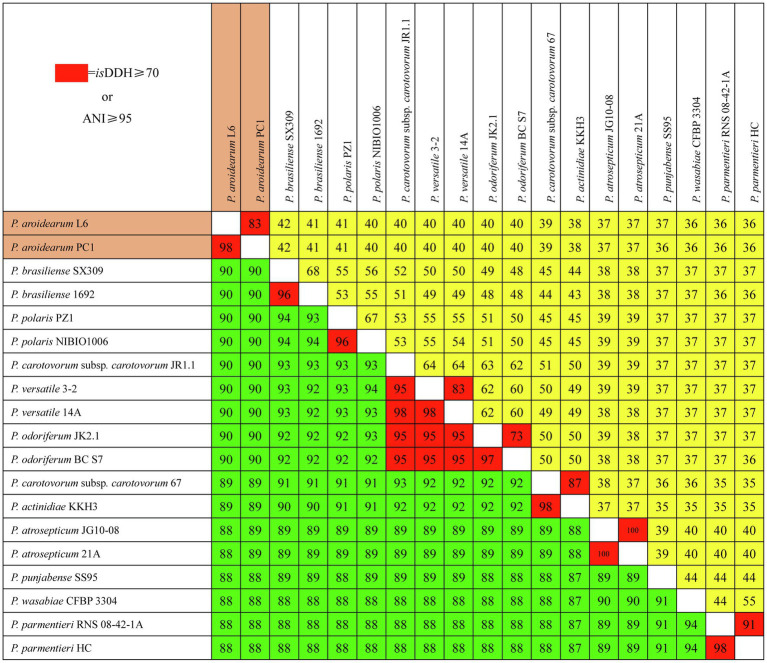
Pairwise comparisons of *is*DDH and ANI values of *Pectobacterium* species. The upper triangle (yellow portion) displays *is*DDH values (%), and the lower triangle (green portion) displays ANI values (%). Boxes with *is*DDH ≥ 70% or ANI ≥ 95% are colored red.

We used *is*DDH and ANI to determine species delineation of *Pectobacterium* spp. Using empirical evidence based on classified species and their comparisons with DDH and ANI values, the same species were set at ≥70% identity in *is*DDH and ≥95% identity in ANI. The *is*DDH and ANI values (Figure 3) were consistent with their phylogenetic relationships (Figure 2), and members of the same phylogenetic clade also showed high *is*DDH and ANI values. The ranges of isDDH and ANI values for 18 *Pectobacterium* strains were 88–100 and 36–100. The *P. aroidearum* L6 and PC1 were evaluated to species level with *is*DDH = 83 and ANI = 98; furthermore, *P. carotovorum* subsp. *carotovorum* JR1.1 and 67 were not the same species with *is*DDH = 51 and ANI = 93, indicating that these two strains were misidentified.

### Analysis of the Core Genome Among *Pectobacterium* Species and Reference Strains

**Figure 4:**
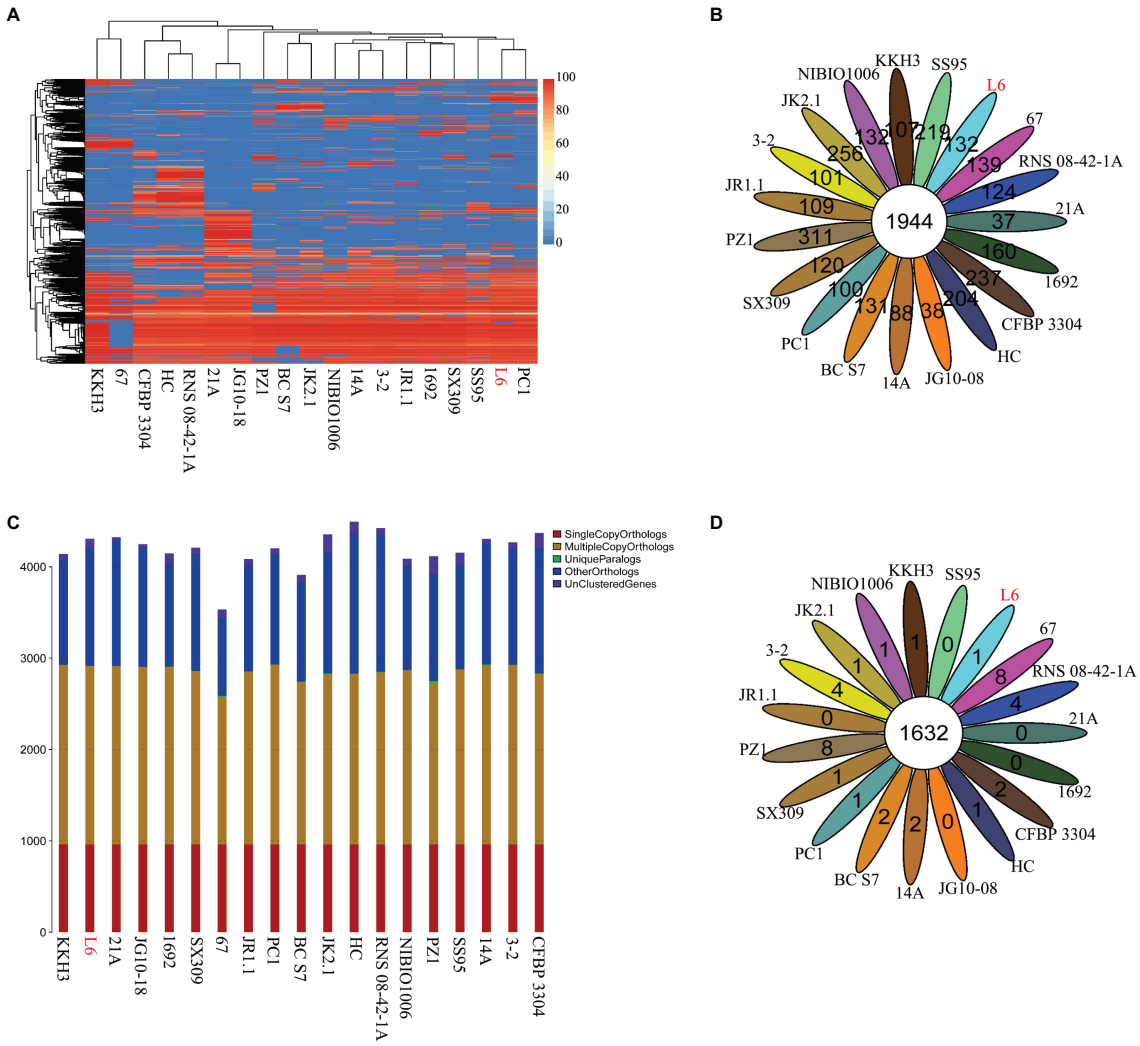
Core genes and dispensable genes and orthologs in the *P. aroidearum* L6 genome and reference strain genomes. **(A)** Dispensable gene heat map (left, dispensable gene cluster; top, strain cluster; red gradient bar represents the scale of similarity percentage); **(B)** Venn diagram of pan genes (each ellipse represents one strain, and the number in the ellipse is the cluster number. One cluster has genes of >50% identity and <0.3 length diversity); **(C)** Ortholog number (Single Copy Orthologs, the number of single-copy homologous genes in the species common gene families; Multiple Copy Orthologs, the number of multiple-copy homologous genes in the species common gene families; Unique Paralogs, genes in specific gene families; Other Orthologs, other genes; and Unclustered Genes, genes that have not been clustered into any families); **(D)** Venn diagram of orthologs in gene family (each ellipse represents one strain, and the number in the ellipse is the family number).

The genomes of 18 *Pectobacterium* strains with *P. aroidearum* L6 were compared. The dispensable gene heatmap showed percentage of dispensable genes among strains. The identity matrix was calculated based on BLASTP. The strains L6 and PC1 had the highest genetic similarity. Otherwise, *P. punjabense* SS95 also grouped together with *P. aroidearum* L6 and PC1 based on the dispensable gene heat map (Figure 4A). Analysis of pan genes among L6 strain and reference strains was carried out. There were 1944 genes shared by all of the bacteria. Among them, 132 genes were unique to L6 and 100 genes were unique to PC1 (Figure 4B). Research on special genes and core genes is important for the detection of functional differences and similarities between samples and provides molecular evidence for phenotype differences and similarities. A gene family is a group of genes that have the same ancestor and comprises more than two gene copies. The members of a gene family have similarity in structure and function, and the proteins produced are also similar. Gene families can be used to detect evolutionary history and gene differentiation. The gene family statistics showed that the final core genome was 1,632 gene families. One gene family was unique to L6, and one gene family was unique to PC1 (Figures 4C,D).

### Analysis of Gene Function Annotation of *P. aroidearum* L6

**Figure 5:**
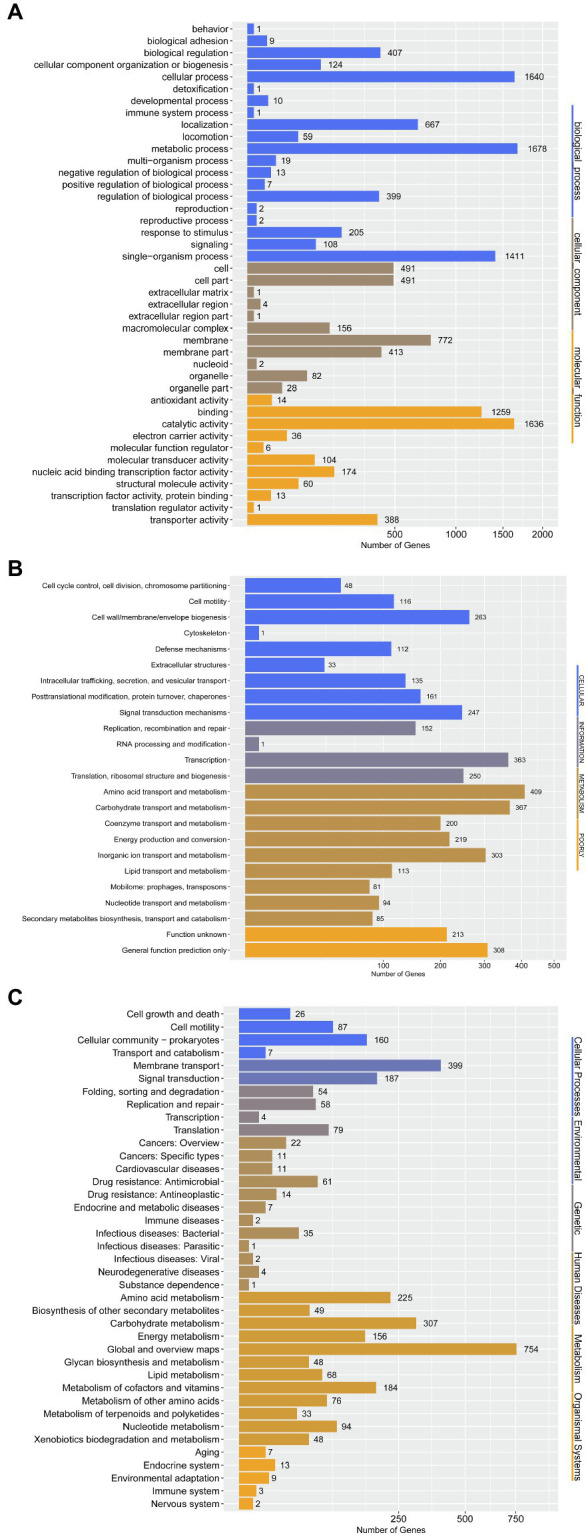
Gene annotation by GO, COG, and KEGG for *P. aroidearum* L6. **(A)** GO function classification of genes in L6. GO analysis was performed for three main categories: cellular components, molecular function, and biological processes. **(B)** COG function classification of genes in L6, grouped into four main parts: metabolism, cellular processes, information, and poorly. **(C)** The KEGG pathway classification of genes in L6 contains six groups: cellular processes, environmental, genetic, human diseases, metabolism, and organismal systems.

To further determine the difference in functions encoded by 4,306 genes of *P. aroidearum* L6, we analyzed the data using GO, COG, and KEGG. A total of 3,013 (65.59%) genes could be annotated to one or more of the GO definitions. In our study, 6,763 genes were annotated to biological processes, 2,441 to cellular components, and 3,691 to molecular functions in GO analysis (Figure 5A). Most gene functions focused on cellular process (1,640), metabolic process (1,678), single-organism process (1,411), membrane (772), binding (1,231), and catalytic activity (1,601). There were 3,625 (78.92%) predicted genes assigned to COG categories (Figure 5B): 43.78% (1,871) of the genes were related to metabolism, 26.11% (1,116) to cellular processes, 17.92% (766) to information, and 12.19% (521) to poorly. A total of 3,081 genes were annotated using the KEGG database (Figure 5C). Among the categories, metabolism was the largest group, containing metabolic pathway (690 genes, 22.40%), biosynthesis of secondary metabolites (321 genes, 10.42%), biosynthesis of antibiotics (234 genes, 7.59%), microbial metabolism in diverse environments (229 genes, 7.43%), and others. The cluster of environmental information processing primarily consisted of ABC transporters (279 genes, 9.06%) and two-component system (173 genes, 5.62%).

### Pathogenic Candidate Genes Obtained Through Gene Annotation Screening

We predicted genes associated with pathogenicity using GO, COG, KEGG, and especially VFDB, PHI, and T3SS, which are important databases for predicting bacterial pathogenicity. The VFDB database mainly focuses on the infectious agents among bacteria, mycoplasma, and chlamydia. A total of 478 (10.40%) genes were annotated to the VFDB definitions. The PHI database contains relationships between pathogens and hosts, and it predicted 432 (9.40%) genes. The T3SS has close relationship with Gram-negative pathogens and aids in determining infection mechanisms and toxicity at the molecular level. There were 723 (15.74%) predicted genes assigned to T3SS categories. Moreover, we screened the relevant genes encoding plant cell-wall degrading enzymes (PCWDEs), toxins, and secretion systems for *P. aroidearum* L6 (Table 3). There were 25 genes related to toxins, and 35 genes encoded PCWDEs, including 28 encoding pectinases, three encoding cellulases, and four encoding proteinases. In addition, 122 genes were involved in six types of secretion systems: 14 genes in Type I (T1SS), 23 in Type II (T2SS), 29 in Type III (T3SS), 25 in Type IV (T4SS), none in Type V (T5SS), and 31 in Type VI (T6SS) secretion systems.

**Table 3:** Pathogenic candidate genes of *P. aroidearum* L6 identified through gene annotation screening.

Type		Number	Gene
Toxins		25	*higB21*, *relE*, *rhaS*, *symE*, *cvpA*, *prtC*, *y4kP*, *higB22*, *hlyC*, *rtxC*, *ortT*, *cbtA*, *abiEii*, *parE1*, *ccdB*, *aebG*, *pasT*, *cptA*, *ccdB*, *yoeB*, *parE3*, *higB23*, *tabA*, *pinD*, *stbE*
PCWDEs	Pectinases	28	*pel1*, *pel2*, *pel3*, *pelB*, *pel4*, *pel5*, *pelL*, *pelW*, *pelP*, *ply1*, *pelD*, *ply2*, *pelX*, *fhaB*, *pehK*, *paxE*, *pehX*, *ogl*, *paaE*, *kduI*, *kdgF*, *plpb*, *fhaB1*, *fhaB2*, *ppbH*, *pglR1*, *ssp., pmeB*
	Cellulases	3	*bcsZ*, *celB*, *exlX*
	Proteinase	4	*btlcP*, *nprE*, *pi38*, *ps53*
Secretion systems	T1SS	14	*lssD*, *lssB*, *lapE*, *cttD*, *hasD*, *hasE*, *hasF*, *aprD*, *prtE*, *prtF*, *tolC*, *lapB*, *lassD*, *mdsABC*
	T2SS	23	*hofQ*, *hofC*, *hofB*, *ppdD*, *pilT*, *gspB*, *gspC*, *gspD*, *gspE*, *gspF*, *gspG*, *gspH*, *gspI*, *gspJ*, *gspK*, *gspL*, *gspM*, *gspN*, *gspO*, *tadC*, *tadB*, *cpaF*, *cpaC*
	T3SS	29	*hrtA*, *ycgR*, *hrtB*, *hrpT*, *hrcC*, *hrtC*, *hrpF*, *fliH*, *hrtD*, *hrcJ*, *hrpB*, *hrpJ*, *hrcV*, *hrpQ*, *hrcN*, *hrtE*, *hrtF*, *hrcQ*, *hrcR*, *hrcS*, *hrcT*, *hrcU*, *fliH*, *fliI*, *fliN*, *flip*, *fliR*, *flhA*, *flhB*
	T4SS	25	*rhsA*, *traC*, *rsmE*, *pilS*, *virB11*, *virB10*, *virB9*, *virB8*, *virB6*, *virB5*, *virB4*, *virB2*, *virB1*, *rhsB*, *pFL4*, *lysM*, *triB*, *trbD*, *trbE*, *trbL*, *ntf2*, *trbG*, *trbl*, *trbK*, *yjgA*
	T5SS	0	
	T6SS	31	*rhsGE*, *hcpA*, *aec32*, *hcpB*, *vgrGA*, *hcpC*, *vgrGB*, *paar1*, *hcpD*, *impB*, *impC*, *iraD*, *vasA*, *vasB*, *vasC*, *vasD*, *vase*, *vasF*, *vasG*, *vasI*, *vasJ*, *vasK*, *vasL*, *hcp1*, *vgrGC*, *paar2*, *hcpE*, *hcpF*, *vgrGD*, *hcpG*, *hcpH*

## Discussion

Plant bacterial soft rot is one of the destructive diseases of cabbage, tomato, and potato (Meng et al., 2017; Wang et al., 2017; Cui et al., 2019). And it always can cause more serious losses than any other bacterial disease (Qi et al., 2021). Most of the soft rot disease of vegetables is caused by *Pectobacterium* spp., one of the top ten bacterial plant pathogens. *Pectobacterium* spp. has attracted more attention about its wide distribution and diversity (He et al., 2021). *Pectobacterium* usually exists in soils with a broad range of hosts; thus, *Pectobacterium* species cause soft rot disease in plants of at least 16 dicotyledonous and 11 monocotyledonous angiosperm families (Ma et al., 2007; Nabhan et al., 2013). *Pectobacterium aroidearum* was classified as a novel species in 2013 (Nabhan et al., 2013). In previous studies, bacterial soft rot disease was caused by *P. aroidearum* in calla (*Zantedeschia aethiopica*; Nabhan et al., 2013), potato (*Solanum tuberosum*; Moretti et al., 2016), Chinese cabbage (*Brassica rapa*; Xie et al., 2017), zucchini (*Cucurbita pepo*; Moraes et al., 2017), konjac (*Amorphophallus konjac*; Sun et al., 2019), pepper (*Capsicum annuum*; Moraes et al., 2020), and carrot (*Daucus carota*; Tang et al., 2021). In our previous research, *P. aroidearum* as a pathogen on *S. podophyllum* was found in China (Xu et al., 2020). It is important to prevent the spread of this pathogen because many ornamental and edible plant species are susceptible to *P. aroidearum*.

The classification of the genus *Pectobacterium* has been subject to wide revision over the last decade. *Pectobacterium* spp. are highly phenotypically, genetically, and pathogenically particularly heterogeneous, indicating a need for re-evaluation and a better classification of these species (Dees et al., 2017). Three subspecies of *P. carotovorum* were reclassified as one subspecies (*P. carotovorum* subsp. *carotovorum*), and *P. carotovorum* subsp. *odoriferum* and *P. carotovorum* subsp*. brasiliense* were reclassified as *P. odoriferum* and *P. brasiliense*, respectively, based on genomics (Liu and Filiatrault, 2020; Liu et al., 2020). A lot of genomes of *Pectobacterium* have been sequenced, annotated, and analyzed previously (Oulghazi et al., 2020; Pedersen et al., 2020; Jonkheer et al., 2021). But there was no information about the whole genome of *P. aroidearum*-type strain SCRI 109. At present, only strain PC1 of *P. aroidearum* has sequenced its whole genome (PC1 formerly classified as *P. carotovorum* subsp. *carotovorum*, the classification has not been corrected in the NCBI database; He et al., 2021). To understand the molecular mechanisms of taxonomy and pathogenic factors in *P. aroidearum*, the whole genome of L6 was successively sequenced in this study. And it will be the first public report on the genome of *P. roidearum*.

Comparison of genomes is an efficient method for classification and detection of bacterial and fungal pathogens (Pritchard et al., 2012; Bühlmann et al., 2013; Malapi-Wight et al., 2016; Tang et al., 2017; Van Dam et al., 2018). Zoledowska et al. (2018) researched the comparative genomic of 15 *P. parmentieri* strains and found the high genomic variation among *P. parmentieri* strains. Pédron et al. (2019) established the taxonomic status of six *Pectobacterium* strains based on phylogenetic data, ANI values, and isDDH results by comparative genomics and identified a novel species of the genus *Pectobacterium* named *Pectobacterium aquaticum*. He et al. (2021) analyzed comparative genomics of four *Pectobacterium* strains and obtained three kinds of highly conserved key pathogenic genes related to cell-wall degrading enzymes in *Pectobacterium* strain PC1, including 19 pectinase genes, 25 cellulase genes, and 22 protease genes. Zhang et al. (2016) compared 85 genomes of the genera *Dickeya* and *Pectobacterium* and found that at least ten tested genomes from these genera were misnamed in GenBank based on ANI, *is*DDH, and whole genome. In our study, L6 and PC1 were grouped in one population distinct from other populations of the *Pectobacterium* genus and we also found some strains were misnamed in GenBank. (*P. carotovorum* subsp. *carotovorum* JR1.1 and 67 were not the same species.) It is effective for re-evaluating current prokaryotic species definition and establishing a unified prokaryotic species definition frame by using whole-genome sequences for taxonomically challenging genera (Zhang et al., 2016; Qi et al., 2021).

Currently, once plants are infected, there is no effective method to control bacterial soft rot (Sun et al., 2019). It is also very possible to develop new control methods. By screening pathogenic genes based on whole-genome sequences of *Pectobacterium* species and analyzing the pathogenic mechanism at the molecular level, Zhang et al. (2017) discovered a total of 168 genes related to pathogenesis including nine specific genes encoding toxins on the genome of *P. atroseptica* JG10-08. Huang et al. (2019) selected five putative effectors from the genome of *P. carotovorum* subsp*. brasiliense* BZA12 and discovered that candidate effector A12GL002483 was localized in the cell nucleus and induced cell death. We discovered 478 genes, 10.4% of total predicted genes, that were potentially related to pathogenesis according to the VFDB database. Previous research has shown that soft rot pathogenesis basically relies on toxins, PCWDEs, and the secretion system. Toxins play a key role in the pathogenicity of *Pectobacterium* species. We discovered 29 genes related to toxins in *P. aroidearum* L6. Moreover, PCWDEs are crucial in three distinct pathogenic functions: degradation, nutrition, and feedback regulation (Franza et al., 2002; Yang et al., 2007). The pathogens benefit from the nutrients produced after degradation; these degradation products accumulate in the host and can induce bacterium to generate more enzymes (Zhang et al., 2017). Therefore, the production of PCWDEs is characteristic of infection by *Pectobacterium* species. The PCWDEs consist of pectinases, cellulases, and proteinases. In this study, we identified a total of 36 genes encoding PCWDEs: 29 encoding pectinases, three encoding cellulases, and four encoding proteinases.

In addition to toxins and PCWDEs, secretion systems play a critical role in plant bacterial disease development. There are six types of secretion systems to export extracellular enzymes and effector proteins in bacteria (Lory, 1998). Through secretion systems, effectors can be transported inside the plant cell and promote rapid infection of the host plant (Holst et al., 1996). Among them, the Hrp gene cluster encoding the T3SS is particular important in many Gram-negative pathogens; this is a multi-protein complex bacterial structure to deliver virulence effector proteins directly into plant cells (Tampakaki et al., 2004; Tam et al., 2007; Xie et al., 2019). In our study, there were 723 predicted genes assigned to T3SS categories. Furthermore, 122 genes were involved in the six types of secretion system in *P. aroidearum* L6 based on GO, COG, and KEGG: 14 in T1SS, 23 in T2SS, 29 in T3SS, 25 in T4SS, and 31 in T6SS. Pasanen et al. (2020) identified a novel species *Pectobacterium parvum* and found it contained SPI-1-type Type III secretion island by comparing between the genomes of *Pectobacterium* species. In the genome of *Pseudomonas syringae*, Kang et al. (2014) researched the role of T3SS effectors in the disruption of actin cytoskeleton and inhibition of endocytosis. In the genome of *Shewanella* sp., Alex and Antunes (2019) detected the genes encoding for T3SS core components and four copies of homologs of T3SS effector. Currently, the effectors of *P. aroidearum* pathogenesis have not been studied. All these genes in *P. aroidearum* L6 have potential virulence functions. Thus, further research on the pathogenic factors in L6 may reveal the mechanism of *Pectobacterium* species infection of plants.

## Conclusion

The classification of the genus *Pectobacterium* has long been unclear. *Pectobacterium* spp. are highly phenotypically, genetically, and pathogenically particularly heterogeneous, can cause severe soft rot in plant hosts, and have a wide host range. Our results suggest that *P. aroidearum* L6 synthesizes and transports virulence factors. Moreover, 182 genes were involved in toxins, PCWDEs, and the secretion system. The results of this research will serve as a foundation for a better understanding of the genomic structure of *P. aroidearum*. The discovery of potential pathogenic factors can help in preventing spread and outbreak of this pathogen and providing effective biological measures against it.

## Data Availability Statement

The datasets used and/or analyzed in the current study are available from the corresponding author on reasonable request. The genome sequence of *P. aroidearum* L6, including all assemblies and annotations, generated for this study is available at NCBI GenBank with accession number CP065044. The 16S ribosomal RNA sequence is available with accession code MT120309.

## Author Contributions

WL and PX designed the experiments and wrote the manuscript. PX, HW, CQ, and ZL performed collection and bioinformatics analysis. CL and WM revised the manuscript. All authors read and approved the final manuscript.

### Conflict of Interest

The authors declare that the research was conducted in the absence of any commercial or financial relationships that could be construed as a potential conflict of interest.

## Footnotes

^1^https://www.bacterio.net/genus/pectobacterium

^2^https://www.ncbi.nlm.nih.gov/genome/?term=Pectobacterium

^3^https://github.com/PacificBiosciences/pbdagcon

^4^https://www.broadinstitute.org/gatk/

^5^http://www.ebi.ac.uk/genomes/plasmid.html; last accessed July 8, 2016

^6^https://www.ncbi.nlm.nih.gov/genome/annotation_prok/

^7^http://tandem.bu.edu/trf/trf.html

^9^http://phast.wishartlab.com/

^10^http://jspecies.ribohost.com/jspeciesws/

^11^http://tygs.dsmz.de/
